# Deep-sea mining rock-fragment dispersal scenarios associated with submesoscale forcings: A case study in the Atlantic

**DOI:** 10.1016/j.heliyon.2024.e34174

**Published:** 2024-07-08

**Authors:** F.N. Amorim, M. Caetano, L. Bastos, I. Iglesias

**Affiliations:** a Interdisciplinary Centre of Marine and Environmental Research (CIIMAR/CIMAR), University of Porto, Portugal; b Instituto Português do Mar e da Atmosfera (IPMA), Portugal; c Department of Geosciences, Environment and Spatial Planning, Faculty of Sciences of the University of Porto (FCUP), Portugal

**Keywords:** Deep-sea circulation, Ocean modelling, Sediment transport, Hazard assessment, NMAR region, ROMS

## Abstract

In the last few years, the exploitation of deep-sea regions for minerals extraction raised international attention as an economically viable source for the mining industry. However, most of these minerals are found close to sensitive ecosystems that can be harmed by mining activities. Given the potential impact, there is a need for the establishment of best practices towards the adoption of preventive strategies for the sustainable management and exploitation of deep-sea environments. To accomplish this objective, numerical models have proven to be reliable tools to support decision-making. In the present study, a high-resolution eddy-resolving ocean numerical model was configured and integrated with a semi-Lagrangian model aiming to map the transport of rock-fragments associated with mining activities. The model was applied to an area rich in polymetallic sulphides at the Northern Mid-Atlantic Ridge (NMAR). Model results showed that local topography and circulation dynamics played an important role for the dispersion and settling of the rock-fragments. In the presence of local submesoscale processes, the residence time do not follow the neutral relation H/vs. It was demonstrated that, depending on the local hydrodynamics, rock-fragments released at sub-surface depth can impact wider areas, with a predicted impact on pelagic and benthic organisms.

## 1 Introduction

The increasing demand for raw materials to meet the modern life needs has directed attention to the potential of deep-sea mining as a possible and economically viable source for mineral extraction. Polymetallic crusts (PMC) on seamounts, polymetallic nodules (PMN) on abyssal plans and polymetallic sulphides (PMS) on mid-ocean ridges can supply raw materials such as copper, nickel, cobalt, iron, manganese and rare earth elements to feed the growing need of the industry [1,2]. However, the majority of these metals are found in mineral resources located close to vulnerable ecosystems [[3], [4], [5], [6]].

The potential hazards associated with deep-sea mining activities are already a concern for the international scientific community, which is committed to understand the impacts of these activities on the deep-sea ecosystems. This is a major goal in several international research projects, such as Blue Mining^1^, MIDAS^2^, Mining Impact^3^, Abyssline^4^, EMINENT^5^, among others. Results from these projects demonstrated that the ecosystems are immediately hit and strongly affected after mining activities. Nevertheless, and despite the importance of deep-sea ecosystems for biotechnology applications, the International Seabed Authority (ISA), at the time of publication of this manuscript, had already granted nineteen exploration contracts to explore PMN^6^, seven to explore PMS^7^ and five to explore Cobalt-rich ferromanganese crusts^8^.

To protect deep-sea ecosystems, ISA has been working on the development of Regional Environmental Management Plans (REMP) [5], designed for a preventive management of deep-sea regions from generalized human activities, and not specifically for mining. ISA Council have adopted the first REMP for the Clarion-Clipperton Zone (CCZ)^9^, a PMN rich abyssal region. Although the CCZ-REMP could be used as an initial standard for the definition of others REMPs, for ridge and seamounts the features and mining techniques may differ from those used at abyssal regions, requiring the evaluation of new considerations based on sound scientific results. Therefore, the Northern Mid-Atlantic Region (NMAR) REMP is still under consideration since the ISA Council is still delineating standardized procedures for the development, review and approval of REMPs.^10^The NMAR presents relevant deposits of PMS interesting for the industry, close to active hydrothermal fields with unique ecosystems^11^ [7]. Although the creation of NMAR-REMP was postponed by ISA Council, its PMS deposits are likely to be commercially explored in the future, since the first lifting test for mining Seafloor Massive Sulphides (SMS) successfully lifted SMS ore from a depth of 1.600 m to the lifting vessel [8,9]. However, the description of this technology does not include the approach that will be selected to eliminate seawater with mining particles which has been raised to the surface jointly with the collected material.

There is a consensus that the major impact of PMS mining at the deep-sea will be loss of substrate, dispersion and sedimentation of possible near floor collecting and return sediment plumes [10], being the size of the fragments present in these plumes greatly dependent on the mining method adopted [[11], [12], [13]]. For instance, modelling efforts to evaluate the environmental impact associated with the Solwara-1 deep-sea mining project, developed to mine mineral-rich hydrothermal vents at the Bismark Sea, considered data from unconsolidated sediments and hard rock particles removed by the collector vehicle [14]. For the modelling of the dispersion and settling of the particles, the average of the classical classification of grain sizes, ranging from <4 μm to 1000 μm, were considered to set the particles size. Other previous works, due to absence of a consensus on the extraction methods, also adopted a similar approach [[15], [16], [17]], but for sites that present at least a thin unconsolidated particle layer or discrete particles at active hydrothermal vents. Therefore, the lack of knowledge about the size of particles generated during mining activities leads scientists to produce models for a wide granulometry range.

Furthermore, it must be also taken into account that the depth of waste discharge is currently not consensual. Three strategies for the return of sediment-laden water are under consideration: i) release as a return plume in the midwater column, typically below the mixed layer depth; ii) a near-bottom release aiming on depositing the sediment in a region that has already been impacted by the collector operations; and iii) send back to the collector vehicle to become part of the collector plume [11,12].

Although there is no consensus of which deep-sea mining technology is the least harmful, among the emerging methods, the commonly proposed is the hydrodynamic suction pick-up mechanism. In this methodology, the collector vehicle will resuspend the bottom sediments and part of it will be discharged in its wake, producing a collector plume. The resuspend sediments kept in the collector will be lifted to the operation vessel and, after being separated from the target fragments, will be subsequently discharged at depth, producing the so-called midwater plume [[11], [12], [13]]. The evolution of both collector and midwater plumes occurs in three different regions, comprising different phases. The initial phase (the near-field region) is controlled by the discharge conditions, characterized by the dominant effect of inertial and turbulent processes in the immediate vicinity of the mining equipment. The second phase (the transition zone) is buoyancy-driven, where the suspended sediment interacts with the surrounding ocean environment. The last phase (the far-field region) is characterized by the passive advection of the plume by local currents, horizontal and vertical diffusion by ambient turbulence, seabed topography constraints, and the settling and deposition of the sediment. By definition, this last phase is concerned with sufficiently low sediment mass concentrations such that buoyancy driven currents no longer plays a dominant role in transporting the sediments [[11], [12], [13],^18^,^19^]. The spatial and temporal scales of the flow dynamics between the near- and far-field regions range from few hundreds of meters to kilometers, and from minutes to several days, respectively [11,18].

The local impacts in the near-field region may be related to sediment redeposition and bottom blanketing, which can potentially bury benthic organisms, clog the respiratory filter feeders and pollute the benthic organisms food supply [20]. The extension of the far-field region is particularly relevant for the quantification of the environmental impact of deep-sea mining sediment plumes since it could harm a wide range of benthic and pelagic organisms [12]. The spatial scales of the impact for both vehicle-generated and midwater return plumes may be on the order of hundreds of square kilometers for nodule habitats, tens of square kilometers for crust habitats and less than tens of square kilometers for vent habitats [21].

In particular, current direction and topography greatly influence the extent of mining impacts in a deep-sea floor massive sulphides [22]. The rugged topography of hydrothermal regions can largely alter the plume extent and residence time of rock-fragments in the water column, depending on sub- to mesoscale features related to topographic constraints or the free passage of currents through flatter regions. Furthermore, changes in ocean currents induced by remotely generated eddies were shown to have great effect on the trajectory and resulting deposition patterns of a deep-sea collector plume [11]. Indeed, observational studies have shown that the circulation at regions deeper than 4 km can become an order of magnitude more energetic in response to the passage of mesoscale eddies, when mining-related plumes are expected to have a wider and faster spreading and dispersion [23].

Therefore, the absent of *in situ* information on the dispersion of rock-fragments from PMS mining, and some limitations of the few previous works [14,16,17], reinforces the needs of dedicated studies for a comprehensive characterization of each hydrothermal region specificities. The topography and depth of each region can considerably change the local and regional circulation patterns, which has a direct effect on the dispersion and spatial extension of the mining-generated rock-fragments. Coupled ocean circulation and Lagrangian models are key tools to provide a reliable representation of ocean dynamics over a wide range of temporal and spatial scales. Hydrodynamic models have been used to simulate complex deep-sea circulation in regions with abruptly changing topography, such as canyons, ridges and seamounts [16,[24], [25], [26], [27], [28], [29], [30], [31]]. These models can reproduce the disturbances in the local density field induced by the morphology of these regions, which contribute to eddy formation, topographically-induced up/downwelling and circulation constraints. The coupling of Lagrangian and hydrodynamic models can account for the impacts of the deep-sea circulation on the behaviour of small elements, including dispersion and recruitment, such as plankton, crustaceans, fish and larvae [[30], [31], [32], [33], [34]], oil spills [35,36], sediments [16,17,37,38] and passive drifters [29]. However, it should be emphasized that oceanic transport forecasting can be highly sensitive to changes in spatial and temporal resolution of the ocean circulation model outputs. For instance, coarser configurations may overestimate the likelihood transport path by averaging out meanders and eddies that would otherwise promote retention [39].

In the light of the above findings, the main objective of the present study is to bring knowledge on how sub- to mesoscale seasonal circulation in the NMAR hydrothermal regions influences the far-field passive advection and settling of rock-fragments from deep-sea mining activities. To accomplish this task we consider i) a realistic eddy-resolving high-resolution ocean model configuration, with nesting capability; ii) a finer temporal and spatial resolution to properly reproduce deep-ocean sub- to mesoscale processes and disturbances in the density field related with topographic constraints; iii) two different seasons to account for the seasonal changes in the oceanic and meteorological forcings; iv) dispersion scenarios close to the bottom and at 200 m from surface to bring knowledge on the rock-fragments dispersion from both the collector vehicle and the midwater return plume below the mixed layer; and v) a settling velocity calculation fitted to the rock-fragment characteristics.

The assumptions made in the present work can be considered an upgrade of previous works already developed in the region [16,17]. With the adoption of a finer model grid resolution that better represents the local bathymetry, the local submesoscale processes can be captured, and, thus, better representing the characteristics of the local circulation. In addition, considering a more realistic settling velocity for the rock-fragments and seasonal scenarios, we are able to depict the differences in the dispersion patterns between seasons.

## 2 Characteristics of the study region

The study region is located in a particular section of the NMAR at the Azores region, where there are ten known hydrothermal active regions, with previous studies about the relationship between benthic invertebrates megafauna and macrofauna conducted in eight of them [7].

**Fig. 1:**
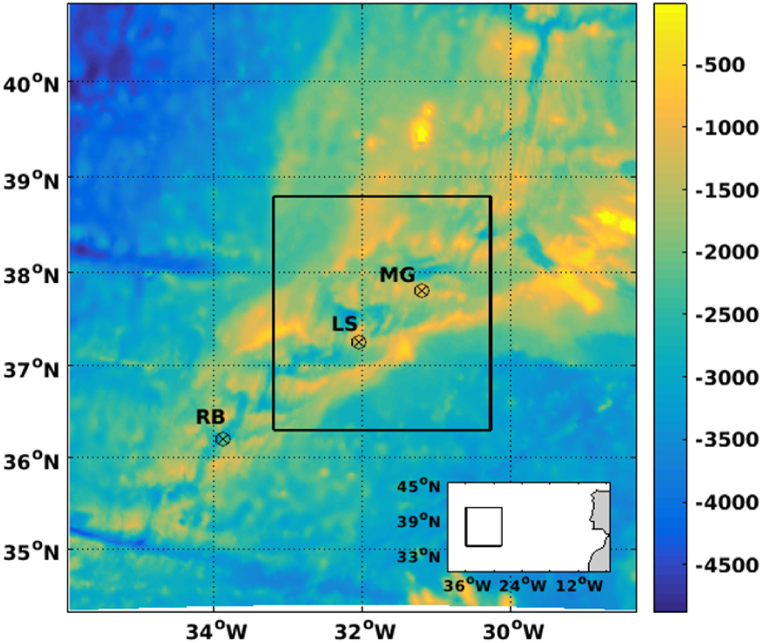
Numerical grid domain. The ⊗ symbols represent the location of the hydrothermal vent areas of Menez Gwen (MG), Lucky Strike (LS) and Rainbow (RB). The bottom box shows the location of the parent grid in relation to the continental Portugal.

Since inactive vent fields are more difficult to find than the active ones, there is a lack of information on the quantity and bathymetric characteristics of inactive fields in the study region. On this basis, we considered as case study areas, the active hydrothermal field regions of Menez Gwen (MG, area of ∼95 km^2^), Luck Strike (LS, area of ∼192 km^2^) and Rainbow (RB, area of about 22 km^2^), with maximum depth of about 850 m, 1740 m and 2320 m, respectively^12^ (Fig. 1). These hydrothermal fields are considered Marine Protected Areas (AMP)^13^, located inside (MG and LS) and outside (RB) of the Portuguese EEZ, which were created for the regulation of human activity in specific areas to achieve conservation or sustainable resource management objectives [40,41].

Observations performed in the NMAR region found peridotite massifs [42] and an upper mantle of peridotites and serpentinized peridotites [43]. High levels of CH4 were measured at LS and MG hydrothermal fluids, related to the serpentinization of the mantle and derived peridotites [44]. Also, the presence of peridotites, serpentinites and steatites was found at the RB hydrothermal vent [[45], [46], [47]]. Due to their mineral composition and commercial interest, the peridotites (density values range 3.1–3.4 g cm^−3^), serpentinites (2.7–3.3 g cm^−3^) and steatites (2.6–2.8 g cm^−3^) are the rocks that could be mined in the study region, producing rock-fragments.

The size of the rock-fragments greatly depend on the cutting technology used and the ressuspension of natural sediments due to the mining procedures adopted. The natural particle sizes of the minerals in the ores depend on the formation processes and can range from 10 μm to 600 μm [48], though larger sizes can form when early-formed minerals are continuously re-crystallized by hydrothermal reworking [49].

## 3 Data and methods

To evaluate the sediment transport and dispersion that might result from deep-sea mining activities in the NMAR region around the Azores archipelago, a Lagrangian model was coupled to the results of a 3D high-resolution eddy-resolving ocean circulation numerical model, implemented to represent the physical oceanographic conditions. A detailed description of the selected numerical models is provided in the following sub-sections.

### 3.1 Regional ocean model configuration

The dynamic behaviour of the ocean on the study region was represented using the Regional Ocean Modelling System - ROMS [50,51], a state-of-the-art versatile 3D model dedicated to represent realistic scenarios, being applied to a wide variety of spatial and temporal scales [[51], [52], [53]]. The model was configured for the NMAR region with a 2-way nesting capability, considering a parent grid with 1/36° (∼3 km) horizontal resolution and a child grid with 1 km horizontal resolution (Fig. 1). Both grids considered 30 vertical levels defined with stretched S-coordinates.

The parent grid was off-line nested into the global ocean eddy-resolving reanalysis product (GLORYS) from the Copernicus Marine Environment Service (CMEMS), with 1/12° horizontal resolution and 50 vertical levels, which provided the initial and daily boundary conditions (temperature, salinity, elevation and velocities). The Generic Length Scale (GLS), fourth-order horizontal advection and third-order upwind advection schemes were used to parameterize the vertical mixing, momentum, and tracers. Along the open boundaries, the 3D ROMS model variables (temperature, salinity and baroclinic currents) were nudged towards the Glorys daily values through a nudging region of 10 grid points (∼30 km), with a spatially variable relaxation time scale. At the open boundaries, the relaxation time scale was defined as τ= 1/3 days (8 h) with a sinusoidal decay inside the nudging layer. The active and passive character of the nudging at the open boundaries are based on the adaptive radiation conditions [54]. The elevation and the 2D velocity are imposed at the open boundaries as a Chapman [55] and Flather [56] boundary conditions, respectively.

Barotropic tidal forcings derived from TPXO datasets [57] were also considered at the parent grid open boundaries. This product provides the diurnal and semi-diurnal components (M_2_, S_2_, N_2_, K_2_, K_1_, O_1_, P_1_ and Q_1_) from the 1/12° regional Atlantic Ocean-ATLAS solution, and the long-period components (M_f_, M_m_) from the 1/4° Global Ocean-ATLAS solution. The child grid was nested online in the parent grid, which provides the contact layer's temperature, salinity, elevation and velocities at each time step. Wind and surface fluxes (humidity, pressure, temperature, precipitation and radiation), derived from the 0.25° and 6-hourly ERA5 Global Climate and Weather Reanalysis Product [58], were internally calculated for both grids using the bulk formulation.

The bathymetry data selected for both grids was obtained through the European Marine Observation and Data Network product (EMODNET), with approximately 200 m resolution. This product was interpolated and smoothed to satisfy the Beckmann-Haidvogel [59] criterion of a maximum topographic stiffness ratio of 0.2. With the vertical mixing scheme adopted, together with the topography parameterization (e.g. high-resolution data and smoothing), horizontal resolution and vertical layer discretization, the model has proven to be able to reproduce deep-sea submesoscale processes related to complex bathymetric systems, as submarine ridges, seamounts and canyons [[26], [27], [28], [29], [30], [31]].

The simulations were ran for 2014 plus a one-month spin-up in December 2013. The year 2014 was chosen to allow the validation of the model solutions with CTD records sampled in the NMAR region, over the LS hydrothermal vent, during July, as part of the Monitoring the Mid-Atlantic Ridge project (MoMar; www.interridge.org). The model results were also validated and evaluated with a set of different observational data: i) absolute dynamic topography and geostrophic velocity derived from AVISO (https://marine.copernicus.eu); ii) thermohaline variability structure derived from several ARGO floats data (https://argo.ucsd.edu/); and iii) seasonal temperature variations along depth derived from the Coriolis-CORA product (www.coriolis.eu.org/Data-Products/Products/CORA).

### 3.2 Semi-Lagrangian model configuration

Lagrangian models can represent a variety of drifters released in the ocean as a number of discrete particles representing a parcel of the total release. These particles can move in an 3D Eulerian field that represents the circulation forcings simulated by numerical hydrodynamic models, ranging from coastal to deep-sea scales [14,16,17,[31], [32], [33], [34], [35], [36], [37], [38]].

The semi-Lagrangian model Ichthyop^14^ was adopted to represent the rock-fragments dispersion patterns that could be associated with deep-sea mining activities in the NMAR area. This model represents the advection and dispersion of discrete fragments of different densities and sizes, using oceanographic fields produced by several numerical ocean models [60], being successful in simulating the dispersion of passive and non-passive particles in deep-sea areas with complex topography [16,29,31].

The 3D velocity (U_p)_ of individual particles is considered as a function of the 3D fluid velocity (U_f_), computed by the ROMS model, a random velocity (u_r_) and the settling velocity (v_s_). Thus, the particle velocity is expressed by the equation [32]:

(1) $$U_{p}\left(x,y,z\right)=U_{f}\left(x,y,z\right)+u_{r}+v_{s}$$

where, u_r_ = δ(2k_h_/Δt)^1/2^ is a random component introduced to the horizontal velocity vector as a function of a real uniform random number δ ∈ [−1, 1] and the imposed explicit Lagrangian horizontal diffusion k_h_ = ε^1/^3 Δx^4/3^. A turbulent dissipation rate of ε = 10^−9^ m^2^ s^−3^ was adopted following previous works that included *in-situ* measurements and numerical modelling [16,32,61–62].

The settling velocity (v_s_) is computed as a function of the sediment particle density (ρ_p_) and diameter (D). Since common size of the rock-fragments that can be generated in potential exploitation of the NMAR region ranges from fine to coarse (Section 2), we adopted the Ferguson-Church formulation [63] to account for the settling velocity:

(2) $$v_{s}=\left(R\;g\;D^{2}\right)/\left(C_{1}\;\nu +{\left(0.75\;C_{2}\;R\;g\;D^{3}\right)}^{1/2}\right)$$

where R = (ρ_p_/ρ_w_) − 1 is the submerged specific gravity, g the acceleration due to gravity, ν the kinematic viscosity of the fluid, C_1_ the constant in Stokes equation for laminar settling and C_2_ the constant asymptotic value of the drag coefficient. With the Ferguson-Church formulation, the resistance imposed by the turbulent drag of the wake behind each grain settling is taken into account, where the viscosity term dominates for small D but becomes negligible for large D. Therefore, for small D the formulation is equivalent to the Stoke's law, while for larger values of D the settling velocity converges to a turbulent drag formulation. With this condition, the formulation can be applicable for particles ranging from very fine, natural grains in the transitional range and very coarse.

**Fig. 2:**
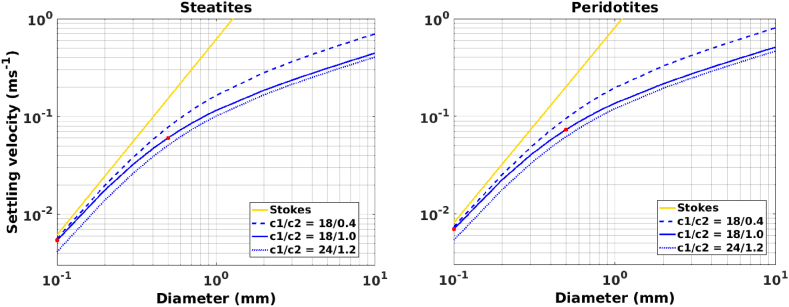
Relation between settling velocity and diameter for particles ranging from fine (D < 0.1 mm) to very coarse (D > 1 mm). For the Ferguson-Church relation (blue lines) we used three pairs of C_1_/C_2_ parameters and for Stokes law (yellow line) C_1_ = 18. The red dots represent the settling velocity value for D = 0.1 mm and D = 0.5 mm adopting the C_1_/C_2_ pair as intermediate relation for grains of varied shape (18/1.0). Modified from Ferguson and Church [63]. (For interpretation of the references to colour in this figure legend, the reader is referred to the Web version of this article.)

The formulation considers three combinations of values for the parameters C_1_ and C_2_, depending on the geometric characteristics of the particles. In this study, the values of 18 (C_1_) and 1 (C_2_) were adopted, which considers an intermediate relation for grains of varied shape, since the rock-fragments that can be generated at the NMAR can range from fine-grained to coarse particles with varied shapes. Fig. 2 shows the results of the Ferguson-Church equation and the Stokes law for the steatites and peridotites, which represent the opposite limits of rock-fragments densities found in the NMAR region (Section 2).

To simplify the calculations, the water density (ρ_w_) was set to a constant value of 1025 kg m^−3^, which is a reasonable approximation considering that the density of the fragments is three times higher than the sea water density. The kinematic viscosity (ν), defined as the relation between the dynamic viscosity (μ) and ρ_w_, was set to 1.36 x 10^−6^ m^2^ s^−1^ for typical water at 10°C and 35 PSU (μ = 0.0014 kg m^−1^ s^−1^). It can be seen in Fig. 2 that for finer sediments the Stokes and Ferguson-Church formulations present close values of the settling velocity, becoming detached toward coarser grain sizes when the drag coefficient is expected to become relevant.

Previous studies on PMS dispersion modelling demonstrated that coarse sands would settle down on the slope near the mining sites, due to the higher settling velocity, while finer particles would spread more widely since their lower fall velocities enable them to be carried by currents before settling [14]. However, as the interest of the industry that are developing machinery for mining PMS is to retain as much ore as possible (raw material) and release a low concentration of particles during the cuttings, the mining tools are designed to minimize the spreading of finer particles [19,48,64,65]. Indeed, an analysis from an *in situ* cutting test using Seafloor Mining Tools (SMT) revealed that the worst-case distribution, based on the maximum plume composition of fine material, presented more than 90% of coarse sand [14].

Since the main objective of the present study is to represent the effect of sub- to mesoscale circulation in the rock-fragments dispersion generated during deep-sea mining activities, in a hydrodynamic region where buoyancy-driven no longer take place (far-field), the modelling tools selected will describe the behaviour of the plumes of larger rock-fragments, including their physical properties (size, density, concentration, roughness). To represent more restricted and wider dispersion scenarios, individual releases of 100 μm and 500 μm peridotites and steatites rock-fragments were chosen, considering the lower limit of the density ranges of these minerals (Section 2) to generate the broader dispersion patterns. These rock-fragment sizes are within the ranges of the particles adopted in similar previous works [14,16,17].

The Ichthyop scenarios covered the boreal summer and winter seasons, to account with intraseasonal changes of the meteoceanographic forcings. For each scenario, 5000 discrete rock-fragments were instantly released over each hydrothermal vent (MG, LS and RB) at two different depths (200 m from surface and 1 m from the seafloor), to simulate the dispersion of both the collector vehicle and midwater return plumes. Hourly Ichthyop simulation results were used to compute each released rock-fragment's maximum traveled distance and the respective travel time.

## 4 Ocean model validation results

### 4.1 Currents

**Fig. 3:**
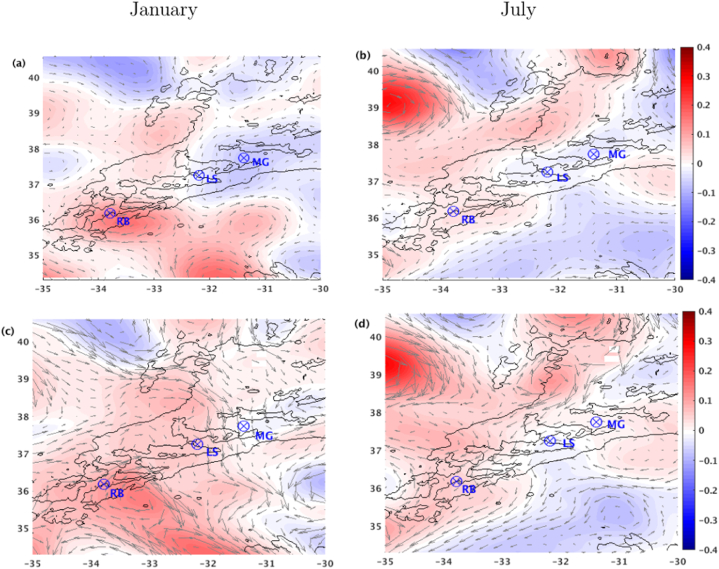
Monthly mean sea level anomalies (m, shaded) and surface geostrophic velocities (m s^−1^, vectors) derived from (a,b) altimetry and (c,d) ROMS results, for the January (left) and July (right). The hydrothermal sites of MG, LS and RB (⊗), and the 2000 m isobath, are presented.

The prominently large-scale feature of the circulation in the study region is the Azores Current (AzC), a long zonal jet meandering at about 34°N [66]. Adjacent to the AzC there is the Azores Countercurrent (AzCC), a subsurface recirculation westward counterflow [67] which together with the AzC occupies an extension of about 2000 m of the upper ocean. To the north and south of the AzC large mesoscale structures can be observed [68,69]. However, Cromwell & Challenor [70] pointed to a weak, but persistent, westward flow around 30°W to the north of the AzC. Those patterns are depicted in the monthly mean surface currents from both AVISO and model results (Fig. 3).

Also, a low dynamic activity to the south of the Azores Islands, in contrast to a higher activity to the north, agrees with the findings of Aguiar et al. [71], based on data from a large set of near-surface drifters. Therefore, the LS and MG vents are located in a region close to the high mesoscale activity, while the mesoscale activities around the RB vent, despite being robust, are more stable.

**Fig. 4:**
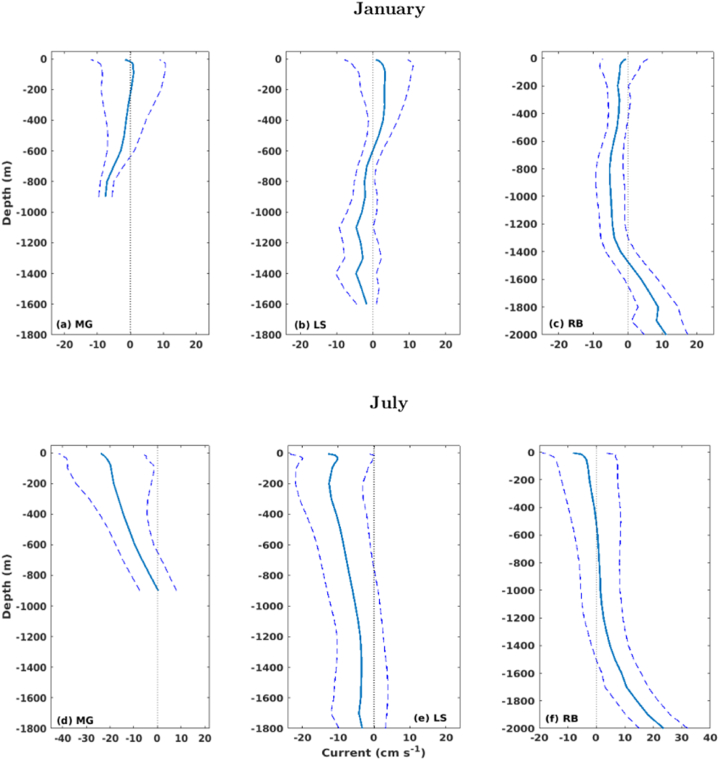
Mean (solid lines) and standard deviations (dashed lines) of modeled zonal currents (u) along depth over the areas of the MG (a,d), LS (b,e) and RB (c,f) hydrothermal fields. Positive (negative) currents are eastwards (westwards).

The mean zonal currents along depth and their standard deviations, derived from model results, for the areas encompassing the MG, LS and RB vents, are presented in Fig. 4. During January, the zonal currents at the MG field (Fig. 4a) showed a weak mean flow from surface to about 500 m depth, with a high variability. This flow became more stable from this depth (500 m-depth) to the bottom. At the LS, the mean zonal current also presented high variability in the first 500 m (Fig. 4b). From this level, it turns westwards and shows a more stable behaviour.

The circulation behaviour observed in the first 500 m in the MG and LS vent sites may be related to the weak but high variable mesoscale activity around these regions during January (Fig. 3a–c). The RB field (Fig. 4c–f) is located in an area where the influence of the AzCC is weak [67,71], resulting in a weak but stable westward flow of about 4 cm s^−1^ from surface to intermediate levels (about 1400 m depth). Below this level, the circulation seems to be influenced by the local topography, turning eastwards and increasing towards the bottom up to 20 cm s^−1^ during July.

The region around the MG and LS vents (Fig. 4d and e) is surrounded by a robust mesoscale activity during July, with the presence of an anti-cyclonic circulation between latitudes 38°N to 41°N (Fig. 3b–d). The circulation at these hydrothermal vents seems to be influenced by the southern border of that anti-cyclonic structure, presenting a high variable and strong westward flow throughout depth, which decreases toward the bottom, more pronounced at MG.

### 4.2 Thermodynamics

**Fig. 5:**
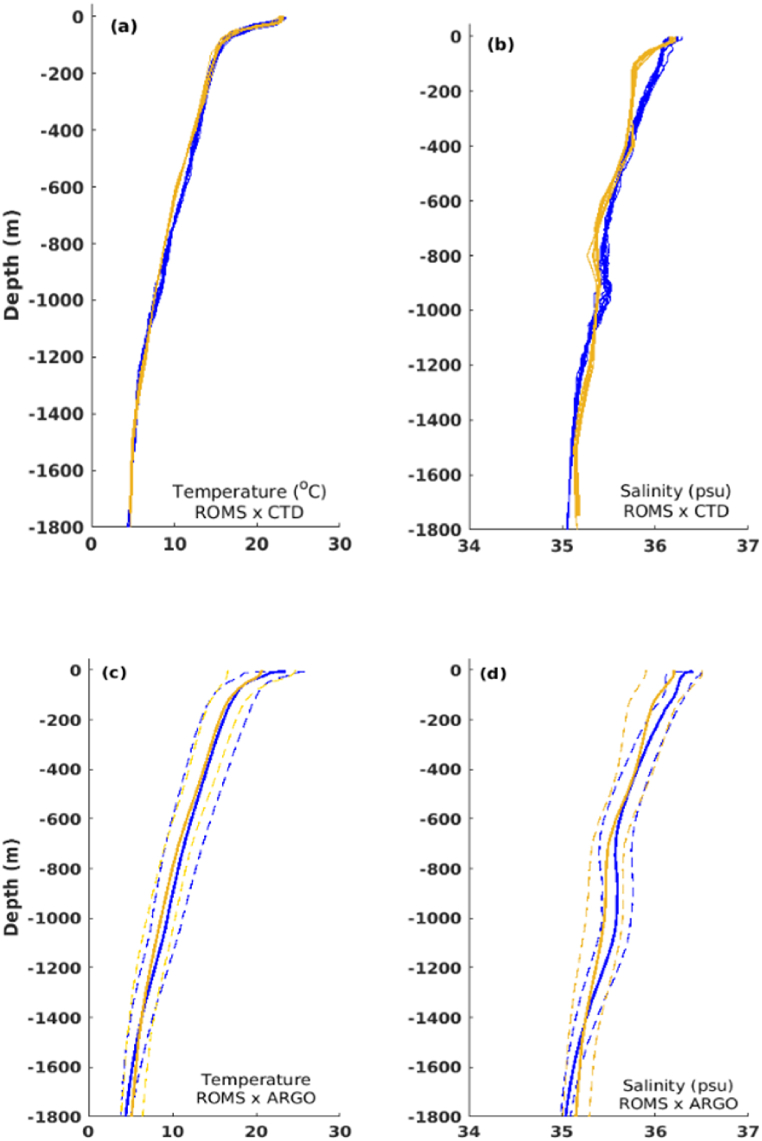
(a,b) Observed (blue) and modeled (yellow) depth profiles of temperature and salinity over LS hydrothermal vent, during July 2014; (c,d) Mean (solid lines) and standard deviations (dashed lines) vertical profiles of temperature and salinity from the model results (yellow) and ARGO floats (blue) found in the region of study during 2014. (For interpretation of the references to colour in this figure legend, the reader is referred to the Web version of this article.)

The capability of the model to reproduce the thermohaline structure was assessed through comparison with MoMar CTD records sampled over the LS hydrothermal vent, during July 2014 (Fig. 5a and b). The modeled temperature profiles are in close agreement with the CTD records, with a good representation of the surface mixed layer observed during July (Fig. 5a). The salinity structure was also well represented by the model, except for a small deviation in the mixed layer (Fig. 5b).

The annual variability of temperature and salinity derived from model output, were compared with several ARGO floats from the study area during 2014. The model results are in good agreement with those obtained from the ARGO data, with the mean values inside the observed standard deviation limits (Fig. 5c and d). The decrease in temperature and salinity values towards the bottom and the decrease in standard deviations were well captured by the model.

**Fig. 6:**
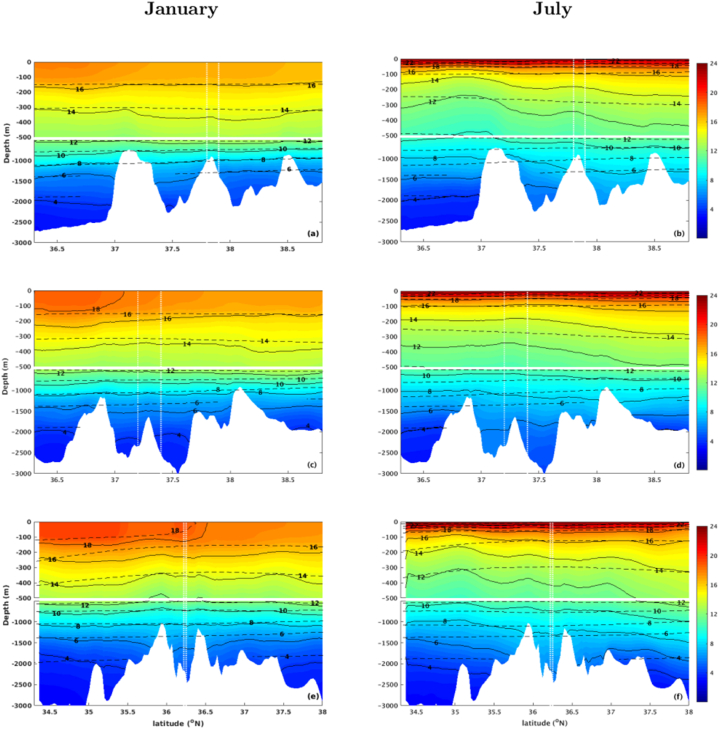
Mean meridional temperature distribution (shaded) along depth at the longitude of the (a,b) MG, (c,d) LS and (e,f) RB hydrothermal fields, derived from ROMS solutions. Black solid (dashed) lines are ROMS (CORA) isothermal contours. Vertical dotted lines represent the latitudinal limits of each hydrothermal field. The bottom topography is represented.

The meridional temperature structure along depth at each hydrothermal site, derived from the model results, is evaluated in comparison with the Coriolis-CORA dataset. The results show different patterns for the winter and summer seasons (Fig. 6). During winter, a surface mixed layer is developed in the first 100 m for all the considered hydrothermal sites (Fig. 6a–c,e), represented by both numerical modelling results and CORA dataset. The subsurface isothermal structure in all vent sites is almost flat during this season, producing a weak mean subsurface flow in the first 500 m at the MG and LS sites (Fig. 4a and b). At the MG (Fig. 6a), a slight rise of the isotherms in the surroundings of the vent site (delimited by the vertical dotted lines) produces a cyclonic circulation, forcing a mean westward circulation, as represented in Fig. 4a. Similar feature can be seen in the RB site (Fig. 6e). In this hydrothermal vent, the instabilities imposed by the abrupt topographic transitions produce a decline of the isotherms and the formation of a local anti-cyclonic circulation, which is responsible for a robust eastward flow below 1400 m depth (Fig. 4c). This disturbance is also represented with the drop of the 6°C isotherm from about 1300 m to 1600 m depth. Despite the good agreement of the depth of isotherms between the model and observed dataset, CORA does not capture the local submesoscale disturbances due to its coarser resolution of 30 km, which reinforces the need of a high-resolution model discretization to capture the local submesoscale features related to topographic constraints.

During the summer season, the surface mixed layer depth is limited to the first 20 m, and the stratified isothermal structure is more affected by the instabilities imposed by topographic changes (Fig. 6b–d,f). The subsurface orientation of the isothermal structures forces a well-developed westward circulation in all hydrothermal sites (Fig. 4d–f). However, while around LS this isothermal structure is somewhat stable along depth, in the surroundings of the MG and RB sites there is a deepening of the isotherms (Fig. 6b,f), which generates an eastward flow (Fig. 4d,f). This flow is intensified toward bottom at RB, due to its deepest topography.

### 4.3 Rock-fragments dispersion

After validated, the ROMS model outputs were used as input to the Ichthyop model.

In this section, we investigate the percentage of rock-fragments deposited along the traveled distance and their respective travel time. We considered that the fragments were deposited when they firstly reach the ocean floor.

**Fig. 7:**
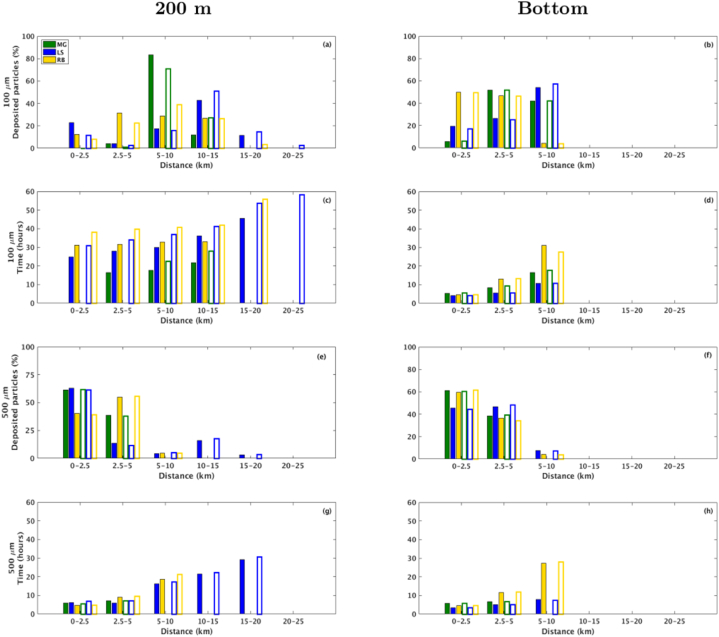
Percentage of rock-fragments deposited (a,b,e,f) over travel distance (km) and their respective travel time (c,d,g,h) for January. The green, blue and yellow bars represent fragments released over the MG, LS and RB hydrothermal vents, respectively. The solid (contoured) bars represent the peridotites (steatites) rock type. (For interpretation of the references to colour in this figure legend, the reader is referred to the Web version of this article.)

**Fig. 8:**
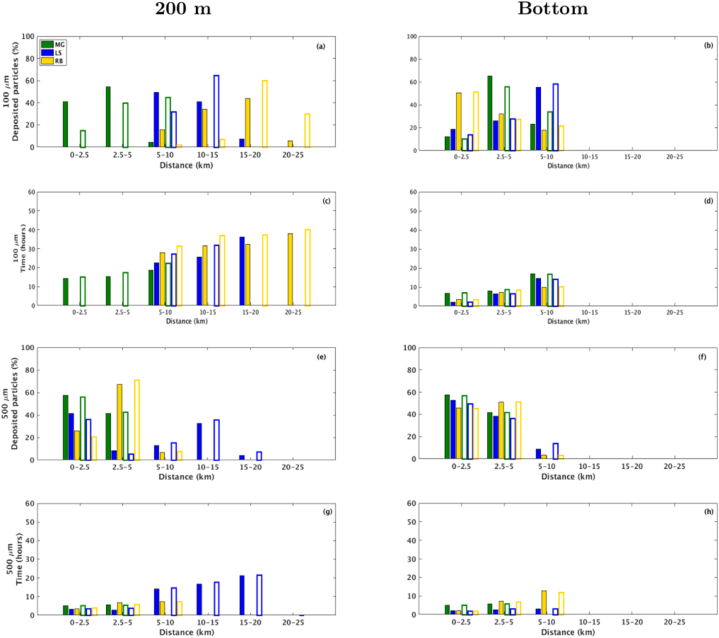
Percentage of rock-fragments deposited (a,b,e,f) over travel distance (km) and their respective travel time (c,d,g,h) for July. The green, blue and yellow bars represent fragments released over the MG, LS and RB hydrothermal vents, respectively. The solid (contoured) bars represent the peridotites (steatites) rock type. (For interpretation of the references to colour in this figure legend, the reader is referred to the Web version of this article.)

Overall, considering the midwater return plume below the mixed layer depth (at 200 m depth), the fragments released at LS tend to travel higher distances and remain suspended for longer periods than those released at MG or RB, although RB is the deepest site (Fig. 7, Fig. 8, left panels).

Fragments with 100 μm size remain suspended for about 50 h until a complete settling, reaching up to 20 km and 15 km at LS and RB, respectively, during January (Fig. 7a–c). During July, fragments released at RB reach as far as 25 km while still suspended for about 40 h (Fig. 8a–c), probably driven by an intense eastward current observed in RB during this period (Fig. 4). At the MG field, considering both scenarios, the 100 μm fragments reach up to 10 km for a complete settling, with an average residence time at the water column of 20 h.

As expected, the 500 μm size fragments released below the photic zone, at 200 m depth, tend to travel shorter distances and remain in suspension for less time than the smaller fragments considered in this study. Results show that releases at MG and RB took up to 9 h for settling more than 80% of both steatites and peridotites rock-fragments, reaching up to 5 km (Fig. 7, Fig. 8), which indicates less dependency on density when compared with the smaller fragments. However, more than 90% of the fragments released at LS can remain suspended for 20 h on average, reaching 10–15 km from the source in about 18 h. The different dynamic behaviour on the considered hydrothermal vents could be related to the bathymetric configuration of each site and local ocean circulation. At LS, the passage of water takes place through rift valleys [72], which may increase the horizontal velocity and turbulence toward the bottom.

Fragments released near the bottom show the tendency to be settled close to each considered site, and more than 90% deposit in few hours (Fig. 7, Fig. 8, right panels). The 100 μm size fragments reach up to 10 km from the source (Fig. 7, Fig. 8b), while most of the 500 μm size fragments were concentrated in the 2.5–5.0 km range (Fig. 7, Fig. 8f).

**Fig. 9:**
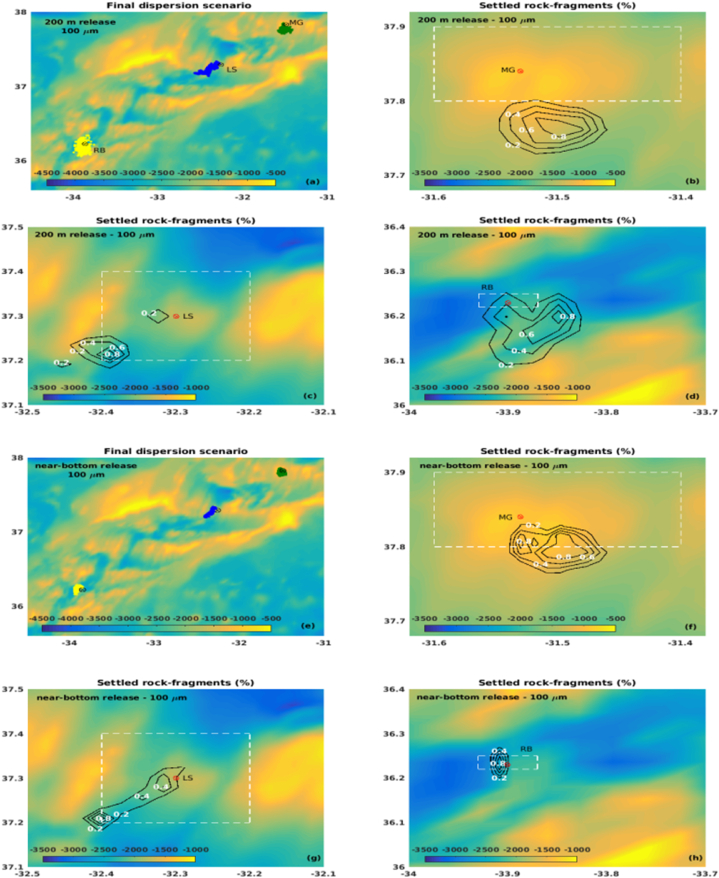
Deposition areas obtained for the 100 μm steatites released at (a) 200 m and (e) near the bottom for January, over the MG (green), LS (blue) and RB (yellow) hydrothermal vents. The percentage of fragments over the settled area are represented by the isolines, within the range 0–1%, each 0.2%. The white-dotted rectangles delimit the AMP areas. (For interpretation of the references to colour in this figure legend, the reader is referred to the Web version of this article.)

**Fig. 10:**
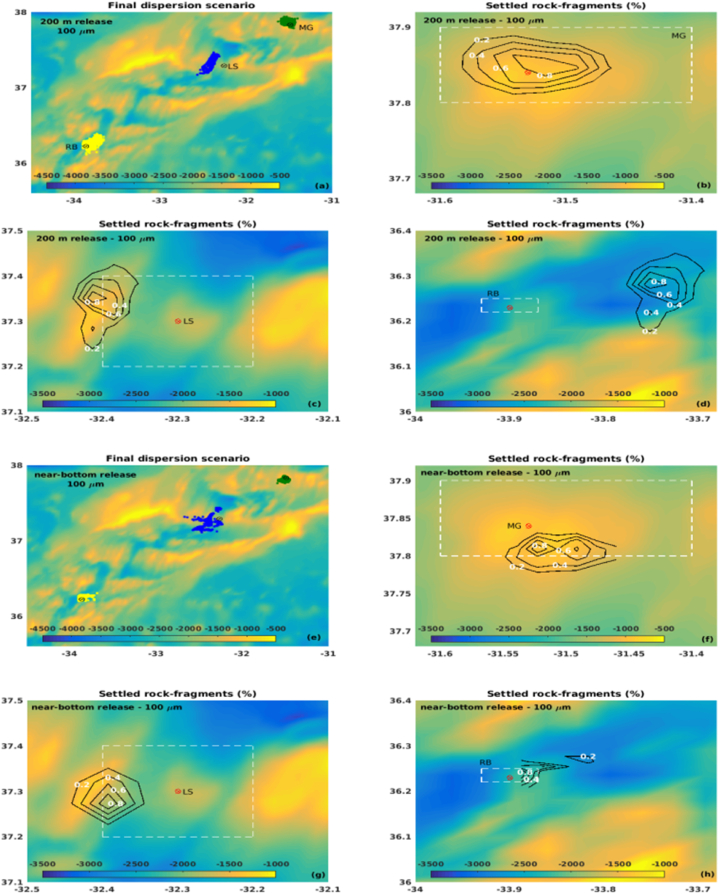
Deposition areas obtained for the 100 μm steatites released at (a) 200 m and (e) near the bottom for July, over the MG (green), LS (blue) and RB (yellow) hydrothermal vents. The percentage of fragments over the settled area are represented by the isolines, within the range 0–1%, each 0.2%. The white-dotted rectangles delimit the AMP areas. (For interpretation of the references to colour in this figure legend, the reader is referred to the Web version of this article.)

Considering the above results, in general, the lighter fragments (100 μm steatites) covered the wider areas for all the considered release sites, being the final positions of the fragments driven by the local circulation and topography. Overall, rock-fragments released at MG were confined closer to the site and within the AMP area, depositing in shallower regions (Fig. 9, Fig. 10b,f), except for the January 200 m release (Fig. 9b), when an increase in the zonal current towards the bottom (Fig. 4a) may have driven the rock-fragments out of the AMP. Fragments released at LS were driven westwards of the site, being scattered over the AMP area during January (Fig. 9c,g) and partly out of the area during July (Fig. 10c,g), probably captured by the more stable westerly zonal current during this season (Fig. 4e). Most of the fragments were settled on the shallower areas that surround the LS hydrothermal vent. At RB, fragments released at 200 m depth were driven eastwards and out of the AMP area, settling in shallower areas during January (Fig. 9d) and deeper areas during July (Fig. 10d), when an intense easterly current was observed below 1800 m depth (Fig. 4f). A similar behaviour could be seen for fragments released close to the bottom (Fig. 9, Fig. 10h).

## 5 Discussion and conclusions

Considering that the *in situ* knowledge of deep-sea conditions and the characterization of its intra- and interannual variability is difficult and expensive, the development of numerical tools to describe the ocean dynamics is fundamental to support safe and sustainable deep-sea exploration and exploitation. After validated, the results of an ocean hydrodynamic model can help in the establishment of guidelines to define deep-sea mining standards in order to mitigate the potential impacts associated with this activity. The coupling of a Lagrangian model to an ocean model outputs can be adopted to simulate deep-sea mining scenarios in order to represent sediment dispersion due different time and spatial forcing scales, ranging from local to regional, and thus providing an early perception of the risk associated with mining activities. In addition, real-time forecasts can give key information that could help to avoid extensive impacts, equipment failures and loss of lives during extreme weather and oceanic conditions.

Hence, the results of a coupled ocean-Lagrangian models can be used to map environmental sensitivity regions and establish safe mining areas, giving key information to regulatory entities to standardize and approve the execution of deep-sea mining activities. Indeed, to address potential impacts from deep-sea mining, the International Seabed Authority (ISA) works on the development of REMP as a good practice to protect deep-sea regions [5].

In the present work, we implemented a nested ocean numerical model for case studies in PMS-rich hydrothermal vent areas, to represent the sub- to mesoscale deep-sea circulation and the features related to topographic constraints and disturbances in the density field. Although active sites cannot be explored, the circulation dynamics are potentially comparable to inactive hydrothermal vent areas. The results of the ocean model were used as input for the semi-Lagrangian model Ichthyop, configured with a formulation that considers rock-fragments with different shape, size and density. Two different seasons were represented to evaluate the effects of seasonal variabilities in the rock-fragments dispersion patterns.

Considering the methodology adopted, which focused on the dynamics of the far-field region of a mining point, the rock-fragments dispersion resulted from the representation of the collector vehicle plume at LS, traveled distances as far as 10 km and remained suspended for about 12 h, while at MG and RB, most of the rock-fragments released close to the bottom can travel as far as 5 km from the release area, remaining suspended up to 10 h. Fragments resulted from the shipboard dewatering released below the photic zone (200 m depth) at MG, the shallower site, remained suspended up to 20 h and traveled as far as 15 km, being settled mainly inside the AMP area. Meanwhile, at LS and RB, fragments released at this depth remained suspended up to 50 h for traveled distances as far as 25 km.

In summary, the fragments spreading patterns observed around the hydrothermal vents area are strongly linked to the local circulation. The residence time of fragments in the water column highlights the influence of local topography on the dynamics of the fragments. The AzC influence at intermediate water depths is weak, being the main transport associated with the AzCC [67,73], which explains the north-westwards/westwards advection pattern of fragments released at MG and LS. The RB region presents a weak AzCC [67,70], being the fragments deposition pattern influenced by the local circulation and the instabilities generated over the NMAR topography. The influence of this topography has effects on the circulation over the entire water column [71].

Model results pointed to higher travel distances for fragments released at 200 m depth, highlighting that midwater return plumes can impact subsurface and bottom ecosystems over large areas. Larger traveled distances were linked to lighter minerals, with a maximum of 25 km for 200 m depth releases and 10 km for bottom releases. The spreading of particulate material has a predicted physical impact on pelagic and benthic organisms by limiting some basic functional activities due to blanketing and abrasion [74]. Furthermore, resuspended PMS particles release high amounts of trace elements (e.g. cobalt, manganese, cooper) to the dissolved phase [74,75]. The release of metals occurs within short time scale reactions (minutes) and keep increasing concentrations with time [75]. This will induce biochemical effects that may lead to dead of organisms (e.g. corals) [74].

The obtained results suggest that for the LS, MG and RB, different areas of exclusion in the NMAR region should be considered in order to define Areas of Particular Interest (APEI), which represents regions protected from human activities and can be designed for a preventive management of deep-sea mining areas. However, a more comprehensive range of factors should be considered for defining important environmental and economic regions, such as intra- and interannual hydrodynamic variability, biota distribution and ecosystem sensitivity to seasonal and interseasonal patterns. Towards that goal, future studies will focus on coupling the oceanic and dispersion models here implemented with ecological models, allowing to evaluate how benthic organisms are impacted by deep-sea mining activities at the whole ecosystem level.

## Data available statement

Data used in the present work, can be freely found at:

CTD records at www.interridge.org

Altimetry at https://marine.copernicus.eu

Coriolis-CORA products at www.coriolis.eu.org/Data-Products/Products/CORA

ARGO floats at https://argo.ucsd.edu/

Regional Ocean Modelling System at https://www.myroms.org/

OSU TPXO Tide Models at https://www.tpxo.net/

Glorys CMEMS global ocean reanalysis product at

https://data.marine.copernicus.eu/product/GLOBAL_MULTIYEAR_PHY_001_030/description

ERA5 Climate Reanalysis product at

https://cds.climate.copernicus.eu/cdsapp#!/dataset/reanalysis-era5-single-levels?tab=overview

Ichthyop Lagrangian tool at https://ichthyop.org/

## CRediT authorship contribution statement

**F.N. Amorim:** Writing – review & editing, Writing – original draft, Visualization, Validation, Software, Methodology, Investigation, Formal analysis, Data curation, Conceptualization. **M. Caetano:** Writing – review & editing, Methodology, Investigation, Funding acquisition, Conceptualization. **L. Bastos:** Writing – review & editing, Validation, Resources, Project administration, Funding acquisition. **I. Iglesias:** Writing – review & editing, Validation, Methodology, Investigation, Funding acquisition, Conceptualization.

## Declaration of competing interest

The authors declare that they have no known competing financial interests or personal relationships that could have appeared to influence the work reported in this paper.

## Footnotes

1 https://www.bluemining.eu.

2 https://www.eu-midas.net.

3 https://miningimpact.geomar.de.

4 http://abyssline.info/.

5 https://www.eminent-project.com/.

6 https://www.isa.org.jm/exploration-contracts/polymetallic-nodules/.

7 https://www.isa.org.jm/exploration-contracts/polymetallic-sulphides/.

8 https://www.isa.org.jm/exploration-contracts/cobalt-rich-ferromanganese-crusts/

9 https://www.isa.org.jm/protection-of-the-marine-environment/regional-environmental-management-plans/ccz/.

10 https://www.isa.org.jm/protection-of-the-marine-environment/regional-environmental-management-plans/northern-mar/.

11 https://vents-data.interridge.org/.

12 https://vents-data.interridge.org.

13 https://www.dgrm.mm.gov.pt/en/web/guest/amp.

14 http://www.ichthyop.org/.

## References

[1] Hein J., Mizell K., Koschinsky A., Conrad T. (2013). Deep-ocean mineral deposits as a source of critical metals for high- and green-technology applications: comparison with land-based resources. Ore Geol. Rev..

[2] NOAA, Ocean explorations and research, accessed. http://www.oceanexplore.noaa.gov.

[3] Van Dover C. (2014). Impacts of anthropogenic disturbances at deep-sea hydrothermal vent ecosystems: a review. Mar. Environ. Res..

[4] Van Dover C., Arnaud-haond S., Gianni M., Helmreich S., Huber J., Jaeckel A., Metaxas A., Pendleton L., Petersen S., Ramirez-Llodra E., Steinberg P., Tunnicliffe V., Yamamoto H. (2018). Scientific rationale and international obligations for protection of active hydrothermal vent ecosystems from deep-sea mining. Mar. Pol..

[5] Dunn D., Van Dover C., Etter R., Smith C., Levin L., Morato T., Colaço A., Dale A., Gebruk A., Gjerde K., Halpin P., Howell K., Johnson D., Perez M., Ribeiros J.A.A., Stuckas H., Weaver P. (2018). A strategy for conservation of biodiversity on mid-ocean ridges from deep-sea mining. Sci. Adv..

[6] Marsh L., Huvenne V., Jones O. (2018). Geomorphological evidence of large vertebrates interacting with the seafloor at abyssal depths in a region designated for deep-sea mining. R. Soc. Open Sci..

[7] Boschen-Rose R., Colaço A. (2021). Northern Mid-Atlantic Ridge hydrothermal habitats: a systematic review of knowledge status for environmental management. Front. Mar. Sci..

[8] Okamoto N., Shiokawa S., Kawano S., Sakurai H., Yamaji N., Kurihara M. (2018). OCEANS-MTS/IEEE Kobe Techno-Oceans (OTO).

[9] Okamoto N., Shiokawa S., Kawano S., Yamaji H., Sakurai N., Kurihara M. (2019). The 29th International Ocean and Polar Engineering Conference.

[10] ECORYS (2014). Study to investigate the state of knowledge of deep sea mining. FWC MARE/2012/06-SCE1/2013/04.

[11] Peacock T., Oullion R. (2023). The fluid mechanics of deep-sea mining. Annu. Rev. Fluid Mech..

[12] Ouillon R., Munõz-Royo C., Alford M., Peacock T. (2022). Advection-diffusion-settling of deep-sea mining sediment plumes. Part 1: midwater plumes. Flowline.

[13] Ouillon R., Munõz-Royo C., Alford M., Peacock T. (2022). Advection-diffusion-settling of deep-sea mining sediment plumes. Part 2: collector plumes. Flowline.

[14] Coffey (2008). Environmental impact statement: solwara-1 project. https://www.yumpu.com/en/document/read/12085050/appendices-5-13-nautilus-cares-nautilus-minerals.

[15] Ortega A., Boomsma W. (2014).

[16] Lopes C., Bastos L., Caetano M., Martins I., Santos M., Iglesias I. (2019). Development of physical modelling tools in support of risk scenarios: a new framework focused on deep-sea mining. Sci. Total Environ..

[17] T. Morato, M. Juliano, C. Pham, M. Carreiro-Silva, L. Martins, A. Colaço, Modelling the dispersion of seafloor massive sulphide mining plumes in the mid Atlantic ridge around the Azores, Front. Mar. Sci. 10.3389/fmars.2022.910940.

[18] Elerian M., Alhaddad S., Helomns R., Van Rhee C. (2021). Near-field analysis of turbidity flows generated by polymetallic nodule mining tools.

[19] Post J., Schreiner V., Weixler L. (2021). The ’vertical approach’ - a sustainable solution for deep-sea mining and sampling. Mining Report.

[20] Gillard B., Purkiani K., Chatzievangelou D., Vink A., Iversen M., Thomsen L. (2019). Physical and hydrodynamic properties of deep sea mining-generated, abyssal sediment plumes in the clarion clipperton fracture zone (eastern-central pacific). Elem. Sci. Anth.

[21] Washburn T., Turner P., Durden J., Jones D., Weaver P., Van Dover C. (2019). Ecological risk assessment for deep-sea mining. Ocean Coast Manag..

[22] Washburn T., Iguchi A., Yamaoka K., Nagao M., Onishi Y., Fukuhara T., Yamamoto Y., Suzuki A. (2023). Impacts of the first deep-sea seafloor massive sulfide mining excavation tests on benthic communities. Mar. Ecol. Prog. Ser..

[23] Aleynik D., Inall M., Dale A., Vink A. (2017). Impact of remotely generated eddies on plume dispersion at abyssal mining sites in the Pacific. Sci. Rep..

[24] She J., Klinck J.M. (2006). Flow near submarine canyon driven by constant winds. J. Geophys. Res..

[25] Kampf J. (2006). Transient wind-driven upwelling in a submarine canyon: a process-oriented modelling study. J. Geophys. Res..

[26] Amorim F.N., Cirano M., Marta-Almeida M., Middleton J.F., Campos E.J.D. (2013). The seasonal circulation of the Eastern Brazilian shelf between 10^o^S and 16^o^S. Continent. Shelf Res..

[27] Aguiar J.A.L., Cirano M., Pereira J., Marta-Almeida M. (2014). Upwelling processes along a western boundary current in the abrolhos–campos region of Brazil. Continent. Shelf Res..

[28] Aguiar A.L., Cirano M., Marta-Almeida M., Lessa G., Valle-Levinson A. (2018). Upwelling processes along the south equatorial current bifurcation region and the salvador canyon (13^o^S), Brazil, Cont. Shelf Res..

[29] Clavel-Henry M., Solé J., Ahumada-Sempoal M., Bahamon N., Briton F., Rottland G., Company J. (2019). Influence of the summer deep-sea circulations on passive drifts among the submarine canyons in the northwestern Mediterranean Sea. Ocean Sci..

[30] Clavel-Henry M., Solé J., Kristiansen T., Bahamon N., Rotllant G., Company J. (2020). Modeled buoyancy of eggs and larvae of the deep-sea shrimp *aristeus antennatus* (crustacea: Decapoda) in the northwestern Mediterranean Sea. PLoS One.

[31] R. Pires, A. Peliz, A. Santos, Into the deep - Dispersal models for deep-water decapod shrimp larvae: The case of *parapenaeus longirostris*, Prog. Oceanogr.,HYPERLINK " 10.1016/j.pocean.2021.102568.

[32] Peliz A., Marchesiello P., Dubert J., Marta-Almeida M., Roy C., Queiroga H. (2007). A study of crab larvae dispersal on the Western Iberian Shelf: physical processes. J. Mar. Syst..

[33] Vaz A., Paris C., Olascoaga M., Kourafalou V., Kang H., Reed J. (2016). The perfect storm: match-mismatch of bio-physical events drives larval reef fish connectivity between Pulley Ridge mesophotic reef and the Florida Keys. Continent. Shelf Res..

[34] Palmas F., Olita A., Addis P., Sorgente R., Sabatini A. (2017). Modelling giant red shrimp larval dispersal in the Sardinian seas: density and connectivity scenarios. Fish. Oceanogr..

[35] Marta-Almeida M., Ruiz-Villarreal M., Pereira J., Otero P., Cirano M., Zhang X., Hetland R. (2013). Efficient tools for marine operational forecast and oil spill tracking. Mar. Pollut. Bull..

[36] Vaz A., Faillettaz R., Paris C. (2021). A coupled Lagrangian-Earth system model for predicting oil photooxidation. Continent. Shelf Res..

[37] Marta-Almeida M., Mendes R., Amorim F., Cirano M., Dias J. (2019). Fundão dam collapse: oceanic dispersion of river doce after the greatest Brazilian environmental accident, mar. Poll. Bull..

[38] M. Marta-Almeida, A. Dalbosco, D. Franco, M. Ruiz-Villarreal, Dynamics of river plumes in the South Brazil Bight and South Brazil, Ocean Dynam.,HYPERLINK " 10.1007/s10236-020-01397-x.

[39] N. Putman, R. He, Tracking the long-distance dispersal of marine organisms: sensitivity to ocean model resolution, J. R. Soc. Interface 10.1098/rsif.2012.0979. PMC3627105 23349437

[40] Menini E., Van-Dover C. (2019). An Atlas of protected hydrothermal vents. Mar. Pol..

[41] Pachiadaki M. (2023). Co-designing a multidisciplinary deep-ocean observing programme at the Mid-Atlantic Ridge in the azores region: a blueprint for synergy in deep ocean research and conservation. J. Mar. Syst..

[42] Fouquet Y., Cambon P., Mevel C., Meyer G., Gente P. (2018). Tectonic setting and mineralogical and geochemical zonation in the snake pit sulfide deposit (mid-atlantic ridge at 23 degrees N). Econ. Geol..

[43] Grácia E., Charlou J., Radford-Knoery J., Parson L. (2000). Non-transform offsets along the mid-atlantic ridge south of the azores (38^o^N- 34^o^N): ultramafic exposures and hosting of hydrothermal vents. Earth Planet Sci. Lett..

[44] Charlou J., Donval J., Douville E., Jean-Baptiste P., Radford-Knoery J., Fouquet Y., Dapoigny A., Stievenard M. (2000). Compared geochemical signatures and the evolution of menez gwen (37^o^50’N) and lucky strike(37^o^17’N) hydrothermal fluids, south of the azores triple junction on the mid-atlantic ridge. Chem. Geol..

[45] Dias A., Barriga F. (2006). Mineralogy and geochemistry of hydrothermal sediments from the serpentinite-hosted saldanha hydrothermal field. 36^o^34’N; 33^o^26’W) at MAR, Mar. Geol..

[46] Dias A., Mills R., Ribeiro da Costa I., Costa R., Taylor R., Cooper M., Barriga F. (2010). Tracing fluid-rock reaction and hydrothermal circulation at the saldanha hydrothermal field. Chem. Geol..

[47] Dias A., Frh-Green G., Bernasconi S., Barriga F. (2011). Geochemistry and stable isotope constraints on high-temperature activity from sediment cores of the saldanha hydrothermal field. Mar. Geol..

[48] Birney K., Griffin A., Kefauver J., Nagai T., Varchol D. (2007). Potential deep-sea mining of seafloor massive sulfides: a case study in Papua New Guinea. Vol. 1, 117 pp, Donald Bren School of Environ. Sci. and Manag..

[49] Herzig P., Petersen S. (2002). Polymetallic Massive Sulphides and Cobalt-Rich Ferromanganese Crusts: Status and Prospects: International Seabed Authority.

[50] Shchepetkin A.F., McWilliams J.C. (2005). The regional oceanic modelling systems (ROMS): a split-explicit, free-surface, topography-following-coordinate oceanic model. Ocean Model..

[51] Haidvogel D.B. (2008). Ocean forecasting in terrain-following coordinates: formulations ans skill assesment of the Regional Ocean modelling System. J. Comput. Phys..

[52] Hilaire A., Vamara K., Philippe V., Christophe L. (2018). Proceedings of CARI 2018. African Conference on Research in Computer Science and Applied Mathematics.

[53] Chakraborty K., Kumar N., Girishkumar M., Gupta G., Ghosh J., Udaya-Bhaskar T., Thangaprakash V. (2019). Assessment of the impact of spatial resolution on ROMS simulated upper-ocean biogeochemistry of the Arabian sea from an operational perspective. J. Operat. Oceanogr..

[54] Marchesiello P., McWilliams J.C., Shchepetkin A. (2001). Open boundary conditions for long-term integration of regional oceanic models. Ocean Model..

[55] Chapman D. (1985). Numerical treatment of cross-shelf open boundaries in a barotropic coastal ocean model. J. Phys. Oceanogr..

[56] Flather R. (1976). A tidal model of the north-west European continental shelf. Mem. Soc. R. Sci. Liege.

[57] Egbert G.D., Erofeeva S.Y. (2022). Efficient inverse modelling of barotropic ocean tides. J. Atmos. Ocean. Technol..

[58] Hersbach H. (2023). Copernicus Climate Change Service (C3S) Climate Data Store (CDS).

[59] Haidvogel D.B., Beckmann A. (1999).

[60] Lett C., Verley P., Mullon C., Parada C., Brochier T., Penven P., Blanke B. (2008). A Lagrangian tool for modelling ichthyoplankton dynamics. Environ. Model. Software.

[61] Tippenhauer S., Dengler M., Fisher T., Kanzow T. (2015). Turbulence and finestructure in a deep ocean channel with sill over flow on the mid-Atlantic ridge. Deep-sea Res. I Oceanogr.

[62] Frère L. (2017). Influence of environmental and anthropogenic factors on the composition, concentration and spatial distribution of microplastics: a case study of the Bay of Brest Brittany, France). Environ. Pollut..

[63] Ferguson R., Church M. (2004). A simple universal equation for grain settling velocity. J. Sediment. Res..

[64] Coffey (2008). https://www.yumpu.com/en/document/read/38646617/environmental-impact-statement-nautilus-cares-nautilus-minerals.

[65] Spagnoli G., Rongau J., Denegre J., Miedema S., Weixler L. (2016). A novel mining approach for seafloor massive sulfide deposits, Offshore Tech. Conference. OTC-26870-MS7.

[66] Klein B., Siedler G. (1989). On the origin of the azores current. J. Geophys. Res..

[67] Comas-Rodrigues I. (2011). The azores current system from a meridional section at 24.5^o^W. J. Geophys. Res..

[68] Pingree R. (1997). The eastern subtropical gyre (north atlantic): flow rings recirculations structures and subduction. J. Mar. Biol. Assoc. U. K..

[69] Sangrà P. (2009). The canary eddy corridor: a major pathway for long-lived eddies in the subtropical north atlantic, deep sea res., Part I: ocean. Res. Papers.

[70] Cromwell D., Challenor P., New A.L. (1996). Persistent westward flow in the Azores Current as seen from altimetry and hydrography. J. Geophys. Res..

[71] Aguiar A., Peliz A., Pires A., Le-Cann B. (2011). Zonal structure of the mean flow and eddies in the Azores Current system. J. Geophys. Res..

[72] St Laurent L., Thurnheer A. (2017). Intense mixing of lower thermocline water on the crest of the Mid-Atlantic Ridge. Nature.

[73] Sala I., Caldeira R., Estrada-Allis S., Froufe E., Couvelard X. (2013). Lagrangian transport pathways in the northeast Atlantic and their environmental impact. Limnol. Oceanogr. Fluid. Environ..

[74] Boschen-Rose R., Colaço A. (2021). Northern Mid-Atlantic Ridge hydrothermal habitats: a systematic review of knowledge status for environmental management. Front. Mar. Sci..

[75] R. Knight, S. Roberts, M. Cooper, Investigating monomineralic and polymineralic reactions during the oxidation of sulphide minerals in seawater: implications for mining seafloor massive sulphide deposits, Appl. Geochem.,HYPERLINK " 10.1016/j.apgeochem.2017.12.027.

